# Supplementary material for: Are carbon emissions trading and green financial instruments synergistic? -Comprehensive quantitative research based on content analysis
Source: PLoS One. 2024 Mar 7;19(3):e0298601. doi: 10.1371/journal.pone.0298601 (PMC10919601; doi:10.1371/journal.pone.0298601)
Supplement: S1 Data — (ZIP) [file pone.0298601.s001.zip › Data/地级市Z_F工作报告.zip!/║┌┴·╜¡/╣■╢√▒⌡╩╨/└┤╫╘╣1⁄2╓┌║┼í╛┬φ┐╦╩2╛▌═°í┐_2007.docx]

各位代表：

　　现在，我代表市人民政府向大会作工作报告，请予审议，并请市政协委员提出意见。

　　一、2003年以来工作回顾

　　2003年以来，在省委、省政府和市委领导下，市政府紧紧依靠全市人民，坚持“努力快发展，全面建小康，振兴哈尔滨”的工作主题，抓住国家实施老工业基地振兴战略的历史性机遇，攻坚克难，创新图强，使改革开放步伐加快，经济实力明显增强，社会全面进步，城乡面貌焕然一新，人民生活显著改善，提前完成了上届人代会确定的各项任务。2006年，实现地区生产总值2094亿元，同比增长13.5%，年均增长13.9%，提前实现在2000年基础上翻一番的目标；财政一般预算总收入192.3亿元，同比增长12.5%，年均增长13%，其中地方财政一般预算收入118.3亿元，同比增长14.7%，年均增长15%；固定资产投资810亿元，同比增长26.7%，年均增长22.4%；社会消费品零售总额894.5亿元，同比增长13.5%，年均增长12.5%。城市综合实力进入全国十强。

　　产业结构形成新格局。三次产业结构由2002年的17.3∶31.9∶50.8调整为2006年的14.9∶36.8∶48.3。工业经济强劲增长，2006年实现工业增加值589.5亿元，年均增长17.4％，对全市经济增长贡献率提高3.9个百分点，机械、食品、医药工业增加值占规模以上工业的66.1％，高新技术产业产值年均增长28.4%。哈药集团、光宇集团、空调股份、松江铜业等企业，利润总额在全国同行业排名第一。亚泰集团哈尔滨水泥公司成为全国水泥行业唯一入选国家循环经济试点企业。农村经济全面发展，2006年实现农林牧渔业总产值476.9亿元，年均增长8.4%。粮食生产连创历史新高，达到98.7亿公斤。无公害、绿色和有机农产品认证面积突破2000万亩。农业产业化规模和覆盖面不断扩大，68%的农户参与产业化经营。新农村建设开局良好，试点村镇面貌有较大改善。第三产业快速发展，2006年实现增加值1011亿元，年均增长13.5%。零售业形成多个新商圈，布局得到优化，业态趋于多样。会展业发展势头强劲。旅游业跻身全国旅游城市竞争力前20名。

　　改革开放实现新突破。国有经济布局战略性调整步伐加快，非公有制经济占GDP的比重达到48.2%。基本完成国有企业改革三年攻坚任务，改制企业1726户，完成职工并轨32.8万人，引入战略投资53亿元，转让国有资产收益38.6亿元。继哈啤、哈药实施资产重组后，量具刃具集团并购德国凯狮公司取得良好效果，得到国务院领导的充分肯定。哈尔滨水泵厂产权转让项目被评为全国“产权交易最佳策划奖”。农村区划调整基本完成，农村税费改革取得重大进展。事业单位改革稳步推进。财政管理体制和投融资体制改革不断深化，市属金融机构改革顺利实施，农村信用社改革全面完成。全方位、多层次、宽领域对外开放格局初步形成。对外贸易较快增长，2006年完成进出口总值27.7亿美元，年均增长12.8%。大型骨干企业自主研发、对外合作和国际市场营销能力不断加强，机电设备、飞机等产品出口大幅度增长，具有自主知识品牌的哈飞汽车出口量位居全国第一。对俄出口加工基地建设态势良好, 对俄贸易稳步增长，科技合作成果显著。2006年实际利用外资4亿美元，引入国内资金155.3亿元，年均分别增长13.1%和46.7%。世界500强企业在哈尔滨市投资项目达到20个。

　　城市发展空间得到新扩展。谋划区域整体振兴，推进“开发江北，两岸繁荣”，先后两次调整行政区划，城市空间得到拓展，市区面积扩大到7086平方公里，是原来的4.3倍。呼兰、松北、哈西、哈东、群力、香坊工业新区、平房工业新区等区域开发建设取得明显成效。哈大齐工业走廊哈尔滨段建设迅速启动，完成投资104亿元，竣工项目51个。

　　城市建设迈出新步伐。四年完成城市建设投资529亿元，新建、改造城市桥梁34座，拓宽、改造城市主次干道及背街土路503条。完成哈肇公路呼兰至通河段建设，通乡通村公路建设成效显著。完成磨盘山供水一期、道里集中供热和沿江污水截流等重大城市基础设施项目。完成市行政办公中心北移，拉动江北开发建设全面提速。治理大气、水污染源1770个，削减污染物1.8万吨。城区新增绿地2425公顷，绿化覆盖率30.2%，实现了创建省级园林城市目标。全市完成造林17.4万公顷，森林覆盖率44.2%。完成太阳岛、中央大街、果戈里大街、沿江风景长廊、犹太会堂、滨江道署等环境综合改造工程，中央大街和太阳岛风景区获得“中国人居环境范例奖”。辟建76个文化和社区广场。新辟、延伸公交线路54条，城区公交站点覆盖率提高3个百分点。市区住宅建设总规模2359万平方米。

　　社会事业取得新进步。科技创新能力增强，2006年有41项产品荣获国家重点新产品，位居东北城市第一、副省级城市第二；技术合同交易额13.64亿元，位居副省级城市第六。教育事业取得新成绩，办学条件明显改善，2003年以来新建、扩建校舍18.4万平方米，改造43所薄弱学校和704所D级危房。卫生事业全面发展，完成公共卫生体系建设项目27个，改造乡镇卫生院47个，新型农村合作医疗试点逐步扩大。人口自然增长率保持在4.3‰以下。文化建设得到加强，群众文化活动不断丰富，城市文化品位进一步提升。体育事业蓬勃发展，成功申办2009年世界大学生冬季运动会，筹备工作进展顺利。精神文明建设取得丰硕成果，成为全国创建文明城市工作先进城市。安全生产工作进一步加强，社会治安综合治理取得成效，信访工作机制不断完善，社会秩序稳定良好。实现国家级双拥模范城“五连冠”。

　　民生状况得到新改善。2006年城市居民人均可支配收入达到11230元，农村居民人均纯收入4405元，年均分别增长12.5%和12.2％。市区人均住房使用面积达到17.54平方米，农村居民人均居住面积达到22.7平方米。就业工作取得进展，累计实现城镇就业51.6万人，城镇登记失业率控制在4%以内。城镇职工养老保险、失业保险和医疗保险覆盖面分别达到86%、88%和73%。城乡社会救助体系进一步完善，累计保障低保对象105.7万人次，救助困难群众132.4万人次。初步建立了农民工工资保障金制度。实施扶贫开发整村推进工程，112个省级贫困村整体脱贫。

　　行政水平有了新提高。坚持依法行政，自觉接受人大依法监督和政协民主监督，人大代表建议、政协委员提案办复率100%。起草审核20件地方性法规草案，制定65件规章和100件规范性文件。行政审批事项由1191项减少到313项。组建市行政服务中心，实行“一条龙”、“窗口式”服务。推进政务公开，政府工作透明度和公共信息服务水平得到提高。廉政建设取得明显成效。妥善应对“非典”疫情、禽流感、松花江水污染等重大突发事件，政府处置突发性公共危机能力经受了考验，得到了提高。

　　各位代表，经过四年的奋发进取、埋头苦干，哈尔滨已经步入全面振兴的快车道，这得益于党中央、国务院、省委、省政府和市委的正确领导，得益于人大、政协和社会各界的监督和大力支持，得益于历届政府奠定的良好基础，得益于全市人民的团结奋斗。在此，我代表市人民政府，向全市人民特别是给予政府工作大力支持的人大代表、政协委员、离退休老同志及各民主党派、工商联、群众团体、无党派爱国人士，向驻哈人民解放军、武警部队，向所有关心和支持哈尔滨发展的港澳台同胞、海外侨胞、国际友人，以及社会各界人士，表示崇高的敬意和衷心的感谢！

　　四年来的奋斗历程使我们深切体会到，推进哈尔滨全面振兴，必须敏锐把握、坚决贯彻党的执政新理念、治国新方略，把全市经济社会运行导入以人为本和科学、自主、和谐发展的轨道；必须密切关注、正确判断国内外发展的大环境、大趋势，调整应变，竞争图强，把哈尔滨的发展融入到国内外发展的大格局中；必须正视哈尔滨经济社会发展相对滞后的现实，坚定不移地把实现又好又快发展作为第一要务，倾全力促发展、谋振兴；必须善于把握政策机遇，采取积极的互动措施，最大限度地发挥国家政策对本地经济社会发展的助推作用；必须始终瞄准哈尔滨未来发展的战略目标，对经济社会发展进行战略设计，抓大事，谋长远，夯实发展基础；必须不断化解影响人民生活和社会和谐的不利因素，调动积极因素，努力保持社会稳定、公共安全和人民安居乐业；必须努力提高行政能力，特别是提高对党的方针政策和市委决策的执行力，转变政府职能，提高抓落实的效力和应对复杂局面的能力。

　　哈尔滨市经济社会发展和政府工作还存在一些必须引起高度重视的问题，主要是：经济运行质量不高，内在动力和增量投入不足，发展后劲不强；经济增长方式比较粗放，资源消耗和环境成本过高，自主创新能力亟待提高；经济外向化程度相对较低，集聚外部资源仍存在体制障碍和环境缺陷；城乡统筹发展机制不完善，农村经济社会发展相对滞后；公共产品供给能力不足，城乡居民就业、就医、就学和住房等基本需求不能得到全面满足；生产安全、环境安全和社会安全存在薄弱环节；政府职能转变不到位，公务员队伍素质和行政效能有待进一步提高。

　　二、关于未来五年和2007年经济社会发展任务的建议

　　未来五年，哈尔滨将进入推动科学发展、促进社会和谐、着力改善民生、加速全面振兴的新时期，既有新任务、新挑战，更有新动力、新机遇，机遇大于挑战。我国综合国力持续提升和全方位开放不断深化，有利于哈尔滨市融入全球经济分工和竞争的进程；中央提出并深入贯彻科学发展观和构建和谐社会战略思想，继续推动老工业基地振兴和粮食主产区加快发展，哈尔滨市将获得更多的政策支持；省委、省政府支持哈尔滨快发展、当龙头，使哈尔滨市区域性中心城市地位和作用更加突出；经过多年改革和发展，哈尔滨市加快发展的内生动力进一步增强；市委确定了建设“适宜创业、适宜人居、适宜人的全面发展”现代文明城市的宏伟目标，将进一步激发全市人民求发展、求富庶、求和谐的热情和干劲。历史性机遇和任务，增强了我们奋发有为的责任感和使命感；全市人民的期待和信任，坚定了我们加快实现全面振兴的决心和信心。我们要坚持以邓小平理论和“三个代表”重要思想为指导，用科学发展观统领经济社会发展全局，加快构建社会主义和谐社会，紧紧围绕建设“三个适宜”现代文明城市的目标，以优化经济结构和转变增长方式为主线，推进改革开放和自主创新，加强资源节约和环境保护，促进社会发展和解决民生问题，着力建设先进制造业、现代农业、现代服务业全面发展的新型产业基地，实现经济、政治、文化、社会建设协调发展，确保完成“十一五”规划的各项任务，加快哈尔滨全面振兴步伐。

　　市十二次党代会确定了建设“三个适宜”现代文明城市和实现哈尔滨全面振兴的宏伟目标，“十一五”规划确定了经济社会发展的具体任务。据此，我们提出五个方面工作建议。

　　第一，加快发展先进制造业和现代服务业。实施工业强市战略，努力建成全国重要的先进制造业基地和新型工业化先行地区。工业增加值占GDP的比重提高到30%以上。坚持以大项目建设带动优势产业发展，做强做大机械制造、高新技术、绿色食品、医药和化工等主导产业，积极培育发展生物医药、新材料、电子信息等战略性产业。以培育高端服务业为重点，促进服务业战略升级，大力培育体验经济服务业集群、知识经济服务业集群、高端要素配置服务业集群。

　　第二，着力推进新农村建设。坚持统筹城乡发展，加快推进农业产业化、县域工业化、农村城镇化，实现农业持续增效、农民持续增收、农村持续发展和农村面貌显著改观。大力发展现代农业，建设优质安全的粮食和畜牧业生产基地。推进农业产业化经营，力争农产品加工业产值与农业产值的比例由0.37:1提高到0.67:1。加强农田水利、农村交通等基础设施建设。建立和完善“工业反哺农业，城市支持农村”的长效机制，推动城市工业和第三产业向农村延伸，鼓励各类资本和人才进入农业和农村。

　　第三，切实加强现代城市功能建设。系统优化城市经济、社会、生态、人居功能，构建产业集聚创新空间、现代城市交通、城市生态工程、居民生活保障和市民休闲空间五大功能体系，使城市环境达到便利、整洁、安全、卫生、适宜生活和创业的良好状态。

　　第四，大力推进文化建设。实施文化立市战略，发挥文化彰显城市个性、提升发展质量的重要作用。整合文化资源，吸收国内外现代文化成果，丰富城市文化内涵，做大做强冰雪文化、音乐文化、金源文化、工业文化、建筑文化等文化品牌。发展公益性文化事业和文化产业，加强城乡文化基础设施建设，满足不同层次文化需求。

　　第五，努力促进民生改善。探索建立促进就业、社会保障、困难群体救助、收入分配调节等长效机制。大力增加教育、卫生等公共产品供给，进一步改善城乡群众就学、就医难状况。

　　2007年，是哈尔滨市经济社会发展一个新的历史起点，是继往开来、续写佳绩的重要一年。经济社会发展主要预期目标是：在节约资源、降低消耗、减少污染的基础上，生产总值增长12.5%，其中工业增加值增长15％；地方财政一般预算收入增长10%；全社会固定资产投资增长25%；社会消费品零售总额增长12.5%；市属外贸进出口总额增长15%，实际直接利用外资额增长15%；城市居民人均可支配收入增长8%，农村居民人均纯收入增长6%；城镇登记失业率4%；人口自然增长率4.5‰；单位生产总值综合能耗降低4%。

　　（一）努力增强工业竞争力，加快新型工业化步伐

　　深入实施老工业基地振兴战略，加快工业结构调整升级，增强工业经济整体实力。

　　加快调整工业空间布局。积极推进区域调整改造，实施呼兰、松北、阿城、哈西、哈东、群力、香坊工业新区、平房工业新区等区域重大基础设施项目建设。推进哈大齐工业走廊哈尔滨段建设，力争35平方公里启动区基础设施建设全面启动，招商引资协议入区项目50项，协议投资额30亿元，30个项目建成投产。整合优化现有开发区、工业园区，强化功能定位，统一招商政策，放大园区带动效应。启动轻型车厂、北方专用车有限公司等企业搬迁改造。

　　大力推进工业项目建设。全年新开工投资超千万元工业项目100个，续建项目260个，竣工投产50个。完成工业固定资产投资260亿元，增长30%。重点推进超大规格特种铝合金板带材、兽用生物制品基地等大项目建设，基本完成煤制烯烃、风力发电等重大项目规划和前期工作。

　　着力缓解工业运行中的突出问题。筹建大项目融资担保公司。依托市股权登记托管中心，开展非上市公司股权质押贷款和股权流动业务。鼓励有条件的企业发行企业债券。引导资金、人才、土地等生产要素向产业集群和工业园区集中，向优势产业和重大项目流动。推行项目集约用地标准，加强闲置土地、园区停建工程的清理和整顿。

　　（二）以发展现代农业为重点，扎实推进新农村建设

　　着力发展现代农业。加快用现代技术和设施装备改造传统农业，提高农业水利化、机械化、信息化、产业化、标准化和规模化水平。优化粮食种植结构，加强优势特色农产品产区建设，打造五常大米等知名品牌。无公害、绿色、有机农产品认证面积扩大到2200万亩。坚持科技兴农，深入实施良种工程、科技入户工程和测土配方工程，推进农业科技示范园区建设。发展壮大畜牧业，推进养殖专业基地建设。创新产业组织形式，推进农业产业化经营，建设一批产业化重点项目。引导发展外向型农业、都市型农业、特色园艺农业和生态农业。

　　壮大县域经济。坚持走主导产业富县、工业经济强县、招商引资兴县、民营经济活县的发展路子，增强县域经济实力。发展农副产品加工业，提升县域产业层次。加强双城、宾县、尚志、五常、延寿、方正等县域开发区建设，推进县域工业融入区域产业分工协作体系。发展劳务经济，推动农村劳动力向二、三产业和域外转移。

　　加强农业基础设施建设。安排农业基础设施建设投资3亿元，其中争取国家和省支持1亿元。实施呼兰河、三家子堤防工程建设，推进西泉眼、友谊、倭肯河、龙凤山、香磨山5个大型灌区配套工程。搞好小流域综合治理，大力改造中低产田。加强农村气象灾害监测预警。探索农业保险等农业生产保护机制。

　　改善农村面貌。建设农村高等级路面公路1000公里，新建沼气工程20处，新增户用沼气池9000个。加快改灶、改厕、改圈和通车、通电、通电话、通电视步伐。扎实推进新农村建设试点工作，编制新农村建设试点规划。采取“逐级申报、总量控制、择优扶持、动态管理”办法，把新农村建设扶持资金投向干部群众积极性高的行政村，重点扶持40个区域中心镇加快发展。

　　（三）突出重点领域，提高服务业水平

　　大力发展旅游会展业。整合旅游资源，加强著名旅游风景区建设，打造近郊文化旅游圈，大力培育冰雪名都、避暑胜地、音乐名城、金源文化、欧陆风情等核心品牌。强化旅游业标准化管理，开展诚信旅游主题活动。加强区域合作，落实东北旅游联合体关于建立无障碍旅游区的协议，拓展旅游市场。发展会展经济，提升哈洽会、冰雪节、哈夏音乐会、哈高科会、啤酒节等知名展会的国际化水平。

　　积极发展金融业。组建产业投资基金管理公司，争取设立地方产业投资基金。推动江海证券成为规范类券商并实现综合性经营。推进中融信托增资扩股。壮大哈投集团，充实民营企业担保基金、中小企业担保基金和“小巨人”企业担保基金，做大小额融资平台。优化金融生态环境，完善社会信用体系。鼓励股份制商业银行和外资银行在哈尔滨市设立机构、开展业务。积极支持企业境内外上市融资。

　　创新发展商贸服务和现代物流业。发展新型商业业态，支持大型批发市场建设和升级。抓好“万村千乡市场工程”试点工作，建立和改造标准化“农家店”600个。整合物流资源，培育龙头企业，发展第三方物流，加快龙运物流园区、华风物流园区、禧龙物流园区、神州物流、新香坊集装箱节点站等大型物流项目建设。

　　有序发展信息服务业。完善网络设施，建设跨领域应用系统，促进信息资源共享。制定服务外包产业发展规划和优惠政策，培育以信息技术和业务流程为重点的服务外包企业，加快市开发区服务外包基地建设。加快电子商务、企业信息化、社区信息化建设，实现市、区、县（市）联动。

　　（四）加强规划、建设和管理，提升城市载体功能

　　强化规划的龙头作用。增强规划的前瞻性、科学性和指导性，发挥规划的统筹、引导和控制作用，提高规划执法力度。做好城市总体规划调整完善工作，完成市区控详规划编制。编制危棚房改造、公园绿地和重点基础设施建设规划。做好哈阿公路沿线绿化及环境整治规划。制定沿江风景带提档升级、何家沟环境整治、老旧楼房综合改造等项目规划。

　　加快城市基础设施建设。启动三环路江南段、道外二十道街跨江桥、何家沟综合整治、磨盘山供水二期和内河清水水源等工程建设。推进和启动滨江热电厂、西南部集中供热工程，加快建设和改造市滑冰馆、冰球馆、八区体育中心、亚布力冰雪运动基地以及奥林匹克主题公园等大冬会工程。推进“三马”地区、老哈尔滨城、沿江地区等综合改造。完成一批“断头路”、“卡脖路”连通改造工程，建设一批达标街路，改造市政土路。

　　加强城市环境综合整治和管理。推进城市景观建设，启动安红街区生态景观廊道建设，借助松花江大顶子山航电枢纽工程下闸蓄水，哈尔滨江段形成高水位的有利条件，续建松花江南岸风景线和临江亲水平台。推进友谊路等临街建筑立面改造。整饰改造红博地下通道。综合改造哈阿、哈黑、哈大、哈五4个出城口区域环境。实施街路、桥体、景观区域及背街背巷灯饰亮化工程。推进数字城管三期工程，完成视频监控系统建设。加强空车配货市场的规范和整治。

　　加强生态环境建设。实施污染物排放总量控制，削减主要污染物3％。启动松花江流域水污染防治规划项目，加强重点企业污染防治和综合治理，推进群力、呼兰、阿城等污水处理项目建设。推动磨盘山饮用水源保护区立法，搞好移民和森林禁伐。加大重点大气污染源、噪声源治理力度。加强城区绿化，植树150万株，新增绿地700公顷。加强生态功能保护区建设，推进退耕还林、防沙治沙、水土保持等工程。开展环保模范城区和生态示范县（市）创建活动，创建省级以上生态示范县（市）1个、环境优美乡镇2个。

　　（五）推动科技进步和资源节约，转变经济增长方式

　　加快自主创新步伐。完善促进企业自主创新的政策和机制，加大科技投入，健全科技创新服务平台。财政支持科技创新的投入达到5亿元，其中市应用技术研究与开发资金1亿元，市发展高新技术产业专项资金4000万元。推动技术研发平台建设，新建3至4个市级工程技术研究中心。完善科技企业孵化平台，新增孵化面积2万平方米。推进技术交易平台建设，做好“哈尔滨国际科技成果展交会”签约项目跟踪服务。加强知识产权保护，推进专利制度建设。支持高新技术研发及其产业化，加快实施重大科技攻关项目，强化产学研战略联盟，推进汽车电子、装备制造、现代医药、新材料等技术研发。加快科技成果转化，大力开发高新技术产品。营造有利于自主创新的人才环境。设立自主创新科技人才专项资金1000万元，支持高新技术领域学科带头人和青年拔尖人才科技创新。鼓励科研机构、高等院校科技人员创办高新技术企业。

　　大力发展循环经济。加快风能、太阳能等清洁能源开发，建设清洁生产和中水回用项目30个。抓好城市和农业节水，推进火电、冶金等高耗水行业节水技术改造。加快推进哈尔滨热电厂、发电厂等企业节能工程，加强采暖期气象节能工作。抓好市开发区、利民开发区两个循环经济工业园区建设。打造医药、食品等行业循环经济产业链。推进全市危险废物处置中心和废弃电子产品回收利用基地建设。

　　（六）深化改革、扩大开放，增强发展动力和活力

　　进一步完善市场经济体制。加快国有企业改革。完善改制企业法人治理结构，推动企业战略重组。妥善处理职工内欠等问题。基本完成尚未改制企业改制工作，对符合条件的企业实施政策性破产和依法破产。推进厂办大集体改革。加强国有资产运营监管，健全企业监事会、业绩考核、财务监督等制度，实行国有资本经营预算和企业经营业绩考核制度。推进供水、供热、供气、公共客运等国有公用企事业单位和房地产开发企业重组改制。放宽市场准入，改善营商环境，引导和鼓励非公有制经济加快发展。加快农村综合改革。推进乡镇机构改革，制定分流人员社会保障、乡镇机构运转经费保障等配套措施。推进“乡财乡用县监管”的县乡财政管理方式改革。推进农村社会事业改革，建立和完善农村社会事业发展经费保障机制。加强税收征管，整顿和规范财税工作秩序，提高公民和企业依法诚信纳税遵从度。完善公共财政制度，深化预算管理改革，扩大财政支出绩效评价和参与式预算试点范围，全面实行市本级国库集中支付制度。深化事业单位改革。

　　推动对外开放升级。加大招商引资力度，充分调动企业的主动性和积极性，做好项目包装，提高签约成功率。加强与国外相关机构合作，建立“招商代表制”，吸引国外行业协会在哈尔滨市设立招商办公室。推动国有及国有控股企业与国外大公司合资合作，力争1至2户企业实现嫁接改造。加强与东北大中城市和沿海地区合作。以“俄罗斯中国年”和“中韩交流年”为契机，加快对俄、对韩出口加工基地、科技合作基地和物流集散中心建设。扩大对外经贸合作领域，拓展与东欧、非洲国家和以色列的贸易往来，优化外贸结构，培育外贸明星企业，打造出口产品名牌。鼓励企业“走出去”，开展劳务输出、境外办厂、技术合作和工程承包。

　　（七）完善公共服务，促进社会和谐

　　实施10项惠民行动：（1）均衡发展教育事业。改善农村办学条件，建设农村寄宿制学校10所，全部免除农村义务教育阶段学生学杂费；扩大职业教育规模，加强职业教育实训基地建设；建立助学机制，保障困难家庭子女接受义务教育。（2）提高医疗卫生服务水平。强化公共卫生体系建设，加大传染病和慢性病防治力度；加强县、乡、村三级医疗卫生网络建设，新型农村合作医疗制度覆盖所有县（市）；市、区两级财政按城区非农业人口每人每年5元标准安排社区公共卫生服务经费，把符合条件的社区卫生服务机构和医疗服务项目纳入基本医疗保险定点和支付范围；启动送医下乡计划，采取定期轮换的办法，从市区医院选派一定数量的医护人员到乡镇和中心村卫生院（所）工作。（3）扩大就业再就业。安置城镇就业再就业11万人，转移农村劳动力117万人。（4）完善社会保障制度。加强社会保险费征管工作。城镇基本养老保险扩大到112万人，失业保险扩大到135万人，医疗保险扩大到180万人，工伤保险扩大到62万人，生育保险扩大到52万人；完善城乡最低生活保障制度；发起建立救助基金，用于救助特困户、农村五保户、灾民及流浪乞讨人员。（5）改善困难家庭住房条件。改造危棚房40万平方米，解决廉租住房700户。（6）加强扶贫开发。推进贫困村新型家园建设，确保62个贫困村整体脱贫。（7）提升社区服务水平。启动建设一批区级社区服务中心和街道社区服务中心。（8）改造建设百座以上城市公厕。（9）改善城市居民休闲、健身环境。建设居民达标庭院500个、市民休闲健身广场50个；中小学校操场和健身设施定时向社区居民开放。（10）改善城市交通。调整、延伸城市公交线路5条，新建景观式候车长廊20处；推进城市中心区与呼兰、阿城等新城区公交连接；加强城市交通环境综合整治，实现交通秩序明显好转。

　　加强和谐文化和社会主义精神文明建设。牢牢把握社会主义先进文化的前进方向，倡导和谐理念，培育和谐精神，建设和谐文化。坚持把社会效益放在首位，推动文化事业和文化产业共同发展，满足城乡居民日益增长的文化需求。深入开展社会主义荣辱观教育，弘扬哈尔滨精神，加强社会公德、职业道德、家庭美德特别是青少年思想道德建设。开展“知荣明耻，信守公德，共建家园”以及“和谐家庭、和谐社区、和谐单位、和谐村镇”创建活动。继续争创国家级双拥模范城。

　　继续做好计划生育、外事侨务、对台、民族宗教、老龄、残疾人事业、国家安全、武装、人防、气象、地震、档案、地方志等工作。

　　三、加强服务型政府建设，提高依法行政水平

　　按照转变职能、权责一致、强化服务、改进管理、提高效能的要求，推进服务和法治政府建设。

　　强化公共服务和社会管理职能。把政府工作重点、政策支持重点和财力保障重点，集中到健全公共服务体系、增加公共产品和发展社会事业上，实现人人共享基本公共服务的目标。建立政府与社会组织分工协作的社会管理机制，健全新型基层社会管理体系。建立科学有效的利益协调机制、诉求表达机制、矛盾调处机制和权益保障机制。加强信访工作，健全社会舆情汇集分析和矛盾排查调处机制，畅通诉求渠道，预防和妥善处理群体性事件。加强应急管理，提高预防和应对公共危机的能力。完善市场监管体系，整顿规范市场经济秩序，加强对食品、药品等关乎民生的重点领域的价格、质量监督检查。强化安全生产管理，加强专项整治，坚决防止重特大安全生产事故发生。加强社会治安综合治理，深入推进“平安哈尔滨”创建工作。

　　深化行政管理体制改革。按照决策、执行、监督相协调的要求，理顺机构设置，优化职能配置，推进政府事务综合改革，探索决策和执行适当分离的行政管理体制。理顺市与区、县（市）的职责分工，推进乡镇综合配套改革。推进政企、政事、政资、政府与中介组织分开。加快行政审批制度改革，规范行政许可行为。改革行政审批方式，探索实施相对集中行政许可权的有效途径和方式，推行网上审批。

　　落实依法行政纲要，建设法治政府。严格按照法定权限和程序行使权力、履行职责、接受监督。贯彻人大及其常委会决议，自觉接受人大依法监督、政协民主监督和人民群众社会监督。加强政府法制工作，完善推进社会事业、健全社会保障、规范社会组织、加强社会管理等方面的法规、规章。规范执法标准和程序，完善评议考核、执法过错责任追究和监督检查等制度。完善行政复议制度和运行机制，调整并加强行政复议机构，健全解决行政争议、化解矛盾的法定机制，引导人民群众以理性合法方式表达利益诉求。坚持严格依法、全面真实、及时便民的原则，推进政务公开。

　　建立政府绩效评估体系，创新政府管理模式。加强政府工作的推进落实，严格责任追究，努力做到各项工作有部署、有检查、见实效，提高工作效能。推进政府绩效管理与评估立法，建立法治化、规范化、科学化的绩效管理体系和标准化、系统化、公开化的绩效评估体系。

　　加强队伍建设，提高公务员素质。强化公务员监督管理，严格作风纪律，规范公务员行为。建立对公务员的投诉、过错追究和质询制度，严格执行政务纪律。按照“为民、务实、清廉”的要求，坚持标本兼治、综合治理、惩防并举、注重预防的方针，推进教育、制度、监督并重的惩治和预防腐败体系建设。培育新型行政文化，引导广大公务员以“八荣八耻”为镜，树立“勤奋好学、学以致用；心系群众、服务人民；真抓实干、务求实效；艰苦奋斗、勤俭节约；顾全大局、令行禁止；发扬民主、团结共事；秉公用权、廉洁从政；生活正派、情趣健康”的良好风气，培养爱民情、责任心、效能感，增强法律意识。加强教育培训，提升公务员履职能力、创新能力和依法行政能力。

　　各位代表，建设“三个适宜”现代文明哈尔滨目标宏伟、任务艰巨，让我们高举邓小平理论和“三个代表”重要思想伟大旗帜，以科学发展观统领经济社会发展全局，深入贯彻省十届人大六次会议和市十二次党代会精神，在省委、省政府和市委领导下，紧紧依靠全市人民，同心同德，励精图治，共同建设哈尔滨和谐美好的未来，以优异成绩迎接党的十七大和省十次党代会胜利召开！
